# Mechanical properties of nucleoprotein complexes determined by nanoindentation spectroscopy

**DOI:** 10.1080/19491034.2020.1816053

**Published:** 2020-09-20

**Authors:** Tatini Rakshit, Daniël P. Melters, Emilios K. Dimitriadis, Yamini Dalal

**Affiliations:** a Laboratory of Receptor Biology and Gene Expression, Center for Cancer Research, National Cancer Institute, NIH, Bethesda, MD, USA; b Department of Chemical, Biological & Macromolecular Sciences, S. N. Bose National Centre for Basic Sciences, Salt Lake, India; c Trans-NIH Shared Resource on Biomedical Engineering and Physical Science, National Cancer Institute, NIH, Bethesda, MD, USA

**Keywords:** Chromatin, nucleosomes, nanoindentation force spectroscopy, elasticity, epigenetics

## Abstract

The interplay between transcription factors, chromatin remodelers, 3-D organization, and mechanical properties of the chromatin fiber controls genome function in eukaryotes. Besides the canonical histones which fold the bulk of the chromatin into nucleosomes, histone variants create distinctive chromatin domains that are thought to regulate transcription, replication, DNA damage repair, and faithful chromosome segregation. Whether histone variants translate distinctive biochemical or biophysical properties to their associated chromatin structures, and whether these properties impact chromatin dynamics as the genome undergoes a multitude of transactions, is an important question in biology. Here, we describe single-molecule nanoindentation tools that we developed specifically to determine the mechanical properties of histone variant nucleosomes and their complexes. These methods join an array of cutting-edge new methods that further our quantitative understanding of the response of chromatin to intrinsic and extrinsic forces which act upon it during biological transactions in the nucleus.

## Introduction

The spatial organization, and mechanical properties of chromatin, the nucleoprotein polymer in living cells, controls DNA accessibility. Chromatin forms the characteristic beads-on-a-string, which is primarily comprised of nucleosomes, which have conserved histone protein constituents, but diverse post-translational modifications (PTM). However, distinct variants of histones exist, and the distribution of nucleosome variants and PTMs is associated with local functional outcomes, such as heterochromatin which is restrictive to RNA polymerases, and transcriptional hotspots which are highly accessible. Biochemical assays and computational modeling have shown that nucleosomes are intrinsically dynamic. Indeed, it has been established that the nucleus exhibits a robust mechanical response because of the elasticity of the chromatin network, which helps balance the mechanical strain of the nuclear lamina [1,2]. This raises the intriguing question whether histone variants can alter mechanical properties of individual nucleosomes, whether such individual mechanical changes propagate through the nucleosome array, and how any emergent properties translate to a biological response. However, studying physical properties of chromatin at the single-molecule resolution *ex vivo* is challenging due to the abundance of nucleosomes and nucleoproteins which gives rise to a crowded environment [3]. Outstanding questions include the rules by which the genome is organized in the nucleus, and whether intrinsically or extrinsically imposed organization regulates its function [4] (or vice versa). Recent work has suggested that chromatin-based mechanics could drive abnormal nuclear morphology and function as well as dysfunction in a variety of diseases [5–7].

In the context of physical forces associated with mitosis, the organization and structure of the kinetochore-associated chromatin have been studied for decades [8,9]. Physical and structural properties of centromeric and pericentromeric chromatin are subject of intense investigations. During chromosome segregation, the mitotic spindles bind to the centromere via the kinetochore. To guarantee proper bi-oriented amphitelic microtubule attachments, the centromeres are subjected to both pulling and pushing forces. Once the spindle checkpoint has been satisfied, chromosomes are swiftly pulled toward the spindle poles. Deciphering whether chromatin contributes structurally to centromere mechanics is a fundamental question in chromosome biology. An immune-electron microscopy dissection of chicken kinetochores in the presence or absence of mitotic pulling forces [10], showed beautifully that the inner kinetochore is greatly distorted in the presence of pulling forces. In the same work, surprisingly, the outer kinetochore was observed to remain static. Another report shows that the yeast centromere provides resilience and pliability under tension during mitosis, behaving as a shock absorber to dampen and dissipate forces generated by the spindle [11,12]. More globally, the effects of histone methylation and acetylation on the mechanical stiffness of mitotic chromosomes were assayed using micropipette-based chromosome length doubling force measurements. In the presence of either histone acetylation inhibitors or histone methylation inhibitors showed that methylation, and not acetylation contributed to mitotic structure and stiffness [13–16]. Recently, an elegant mathematical model was developed which predicted that microtubule configuration-dependent phosphorylation of the kinetochore is tension-dependent [17]. Indeed, during chromosome segregation, these authors found that the chromosome passenger complex must interact with the microtubules in order to efficiently phosphorylate the kinetochore [17]. Taken together, these reports support the possibility that mechanical properties of centromere chromatin directly impact biological functions.

Recently, our group has interrogated the mechanical properties of centromere-specific CENP-A nucleosomes and extended the work to understand how such properties can be modulated by its conserved and essential binding partner CENP-C using a nanoindentation force spectroscopy method and computational simulation [18,19].

Herein, we describe our adaptation of in-fluid single-molecule nanoindentation force spectroscopy to determine the Young’s modulus of nucleoprotein complexes. Previous nano-elasticity measurements have been performed on various biological systems, ranging from bones to macromolecular complexes [19–27]. In this manuscript, we explain how to apply our modified protocols to recombinant *in vitro* reconstituted nucleosomal complexes or to chromatin complexes purified from human cells. We believe this protocol is useful to the field because it can be used to probe biomechanical properties of variant and modified nucleosomes in highly controlled *in vitro* settings, as well as to chromatin extracted from cells.

### AFM nano-elasticity as a tool to probe molecular mechanics

With pico-newton force sensitivity and sub-nanometer displacement accuracy, atomic force microscopy (AFM) is a useful tool for measuring the elastic moduli of biological samples using nanoindentation exerted by the AFM tip [26,27]. The primary advantages of AFM nanoindentation are the simultaneous ability to determine topography at nanoscale resolution and measuring effective elasticity at a precise location of a biomolecule. Moreover, the ability to study systems in real-time and *in vitro* under physiological conditions are major assets in the determination of nanomechanical properties of biological molecules.

## Methodology

AFM samples are prepared usually by the droplet cast method [28,29] on muscovite mica since it offers atomically flat surface. Depending on the type of application, gold on mica [23,30–32] substrates is occasionally used. The sample should be properly adhered to the underlying substrate by electrostatic attraction or by covalent binding in order to withstand the raster scanning by the cantilever. Usually, silicon nitride cantilevers with spring constant in the range of 0.01 N/m to 0.5 N/m are used for AFM biological applications in fluid in order to prevent damage to the sample. This protocol is standardized for Asylum research Cypher S and Bruker Multimode 8 AFM instruments, but it should be easily adaptable to other commercially acquired or custom-built AFM systems.

In addition to imaging the topography of samples, another major application of AFM is force spectroscopy. Force spectroscopy involves the direct measurement of forces between the tip and the sample surface as a function of the distance between the two. The horizontal axis of a force curve represents the relative vertical distance movement between the sample and the AFM probe, and the vertical axis is the deflection of the cantilever as the tip is moved toward the sample surface, contacts, and pushes against the surface and then away from the surface.

In an AFM force measurement experiment, the sample is moved in the vertical direction relative to the AFM probe, by applying a voltage to a piezoelectric translator and the cantilever deflection is measured. To obtain a force–distance plot, two simultaneous events are measured – the relative movement of the AFM cantilever toward (approach) and away (retract) from the sample and the cantilever deflection ΔZc. The force F is obtained by multiplying the deflection of the cantilever with its spring constant. The tip-sample force is described by Hooke’s law F = −KcΔZc (eqn 1), where Kc denotes the spring constant of the cantilever [26]. For nanoindentation experiments, the cantilever is pushed onto the sample surface applying a force in the range of a few tens to hundreds of nano-newtons, depending on the cantilever stiffness. The sample responds to indentation according to its viscoelastic properties and the acquired force–displacement curve (which translates into force-indentation data) can be fitted with appropriate contact mechanic models to extract mechanical parameters. The shape of the probe is a critical parameter in all contact mechanic models. Various theoretical and empirical models have been developed to analyze the force–displacement curves [33–35]. All such models stem from the original work by Hertz, who first analyzed the contact problem between two spheres [36,37].

In the Hertz model, the adhesion of the sample is neglected; therefore, it can be applied when the adhesion force is much smaller than the maximum load applied. Furthermore, it is assumed that the indenter is not deformable and there is no additional interaction between the sample of interest and the indenter. In the study of soft materials, the Hertz model predicts that indentation with a cone or a sphere, with the loading force (F) as a function of the indentation (δ), can be expressed by, F(cone) = π/2E(1 − ν^2^)tan(α)δ^2^ (eqn 2) and F(sphere) = 4/3E(1 − ν^2^)R^1/2^δ^3/2^ (eqn 3), respectively. The precise geometry of the indenter up to the maximum indentation depth determines which equation is to be used. In these equations, E is the elastic modulus, ν is the Poisson’s ratio, δ is the indentation, α is the opening angle of the cone, and R is the radius of the sphere. The Poisson’s ratio (ν) is generally set to be 0.5 as most biological samples are near incompressible [23]. The value of Poisson’s ratio is the ratio of transverse expansion to axial compression. Most materials have Poisson’s ratio values ranging between 0 and 0.5. The original Hertz model considers the contact between two spherical bodies but several extensions for different indenter geometries were attempted later [26]. The Hertz model assumes that the indentation of the sample is small in comparison to the sample thickness. Thus, indentation depth has to be optimized by modulating the applied force. Some of the most commonly used models (Hertz, DMT, JKR, Oliver-Pharr, etc.) for elastic modulus determination are usually provided with the inbuilt AFM analysis software and the user instructions are available in the software manual.

## Materials

### Nucleosome reconstitution by salt dialysis

- Slide A lyser 20 K MWCO (Thermo Fischer) Dialysis cassette
- Syringe with needle (20 Gauge)
- magnetic beads and magnetic stirrer
- Amicon Ultra-4 Centrifugal Filter Unit with Ultracel-3 membrane (Millipore)
- Chromatin dialysis buffer solutions: 2 M NaCl, 10 mM Tris-Cl pH 8.0, 1 mM EDTA; 1 M NaCl, 10 mM Tris-CL pH 8.0, 1 mM EDTA; 0.8 M NaCl, 10 mM Tris-CL pH 8.0, 1 mM EDTA; 0.6 M NaCl, 10 mM Tris-CL pH 8.0, 1 mM EDTA; 0.15 M NaCl, 10 mM Tris-CL pH 8.0, 1 mM EDTA. All buffers should be made under sterile conditions, and kept ice-cold.
- H2A-H2B dimer, H3/H4 tetramers, CENP-A/H4 tetramers (EpiCypher, Research Triangle Park, NC).
- 601 DNA plasmid (pGEM3Z-601 from Addgene) in 10 mM Tris-Cl pH 8.0 buffer, 0.2 mg/ml
- 187-bp 601 sequence (EpiCypher, Research Triangle Park, NC)
- Protease inhibitor cocktail (complete, Roche)

### *List of materials for* ex vivo *sample preparation*

- HeLa cells
- DMEM (Invitrogen/ThermoFisher Cat #11965) supplemented with 10% FBS and 1X penicillin and streptomycin cocktail.
- MNase/mL (Sigma-Aldrich cat #N3755-500UN)
- CaCl_2_ (Sigma-Aldrich cat #449709)
- EGTA (KD Medical cat #PMB-0700)
- EDTA (Quality Biological, Inc cat #351-027-721)
- PBS (KD Medical cat #RGF-3210)
- Protease inhibitor cocktail (Roche cat #05056489001)
- ACA serum (BBI Solution cat #SG140-2)
- Anti-CENP-C (MBL International cat #PD030)
- Protein G Sepharose beads (GE Healthcare cat #17-0618-02)
- Tabletop centrifuge
- End-over-end rotator

### AFM supplies

- 1-(3-aminopropyl) silatrane APS (for mica functionalization methods see ref. 26)
- Muscovite mica, grade V1 (SPI supplies, PA, USA).
- Molecular biology grade PBS buffer pH 7.4
- 2 mM MgCl_2_
- AFM scanning system (we use Oxford Instruments, Asylum Research’s Cypher S AFM, Santa Barbara, CA or Bruker Multimode 8 AFM)
- Olympus/Bruker Biolever mini (spring constant 0.1 N/m) and MSNL-E with nominal stiffness of 0.1 nN/nm, Bruker
- Asylum Research software version 15 and later and Bruker Nanoscope Analysis v.1.9 and later
- Origin 8.0 software

## Methods

### Nucleosome reconstitution

We use the classical Stein and Simpson salt dialysis protocol to reproducibly reconstitute high-quality nucleosomes [38,39] under close to physiological conditions (150 mM NaCl, 2 mM MgCl_2_) with the following adaptions refined over the last decade in our lab [40,41]. We have tested other reconstitution protocols, and find that NAP-1/chaperone-mediated assembly is equally effective at getting fully wrapped octameric particles as measured by AFM and by native PAGE.

**Figure 1.**
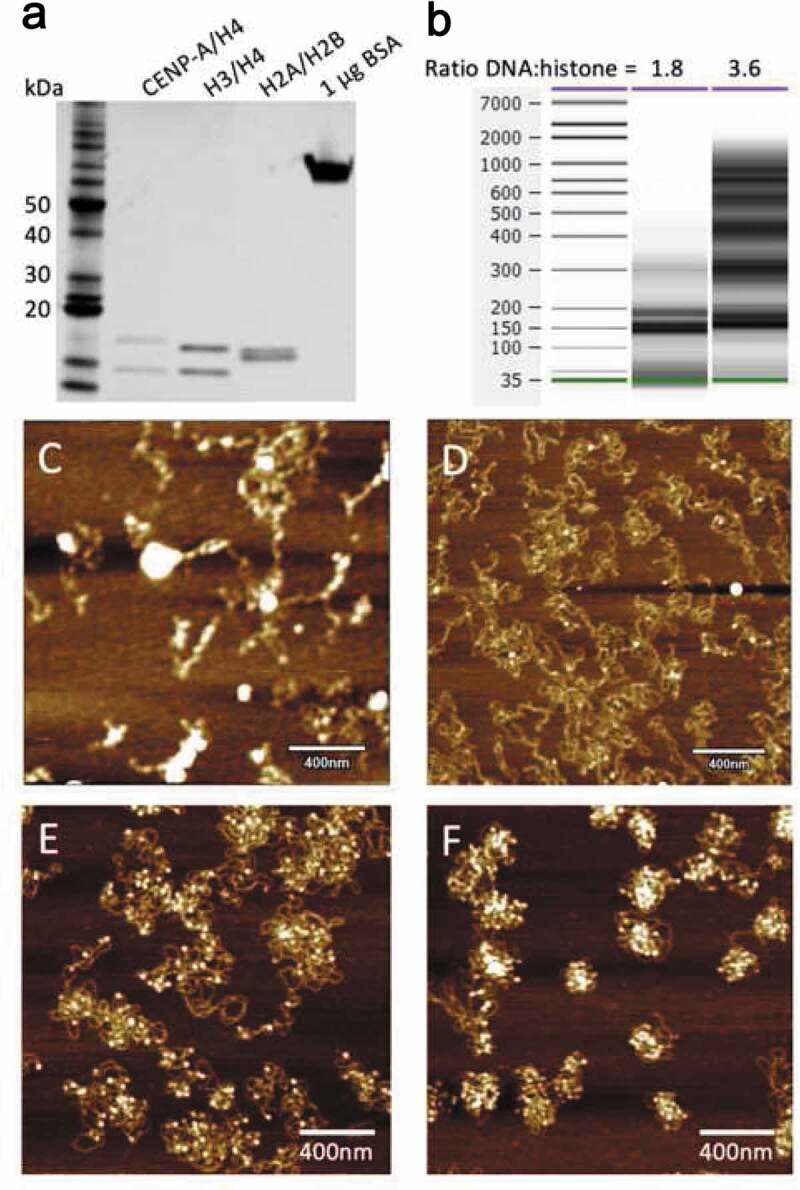
(a) Histone protein concentration was determined by Coomassie staining using a BSA standard. (b) BioAnalyzer results from a 60 sec. MNase digestion of reconstituted H3 with different ratios of DNA to histones, showing a nucleosome ladder pattern. AFM images of (c) frozen samples, (d) poor quality reconstitution, and good quality reconstitution with either a DNA:histone ratio of (e) 1.8 or (f) 3.6.

1. All surfaces are sterilized with 70% ethanol prior to reconstitution, glassware should be meticulously cleaned and dried with no traces of soaps or surfactants. Autoclaved pipette tips and glassware are critical to prevent contamination from nucleases and proteases. We suggest maintaining a single-taped off ‘clean’ bench area dedicated for reconstitutions. Milli-Q water was used for all solution and buffer preparations. All buffer components were procured from Sigma Aldrich. We check the plasmid DNA quality on agarose gel after each plasmid preparation. We recommend avoiding the use of nicked plasmids as the best quality reconstitutions are obtained on supercoiled plasmids. The concentration of the DNA solution is measured by UV-spectroscopy before storing them at −20°C; and 20–50 µg aliquot of histones in 2 M NaCl buffer should be gently thawed on ice, 30 min before use. For re-use, histone aliquots can be stored at 4°C but must be disposed within 2 weeks if not used, and if they do not contain glycerol. After thawing histone aliquots, dialyze H3/H4 tetramers or CENP-A/H4 tetramers with gentle stirring against 1 L pre-chilled 0.1 M, 0.6 M, and 2 M NaCl-TE, respectively, for 1 hour. For dialyzing the histones, the volume should not be more than 200 µl. For a 40 µg reconstitution, take 9 µg of H3/H4 tetramers or CENP-A/H4 tetramers. Measure the concentration of H2A-H2B dimer and dialyzed H3/H4 tetramer or CENP-A/H4 tetramers by PAGE analysis using 1 µg of BSA as a standard, followed by Coomassie staining (Figure 1(a)). By measuring the band intensity of the histone proteins over BSA, the concentration of histone proteins was determined.

[Note 1: This step is important, since after dialysis, there will be some loss of histones, measuring the concentration of the histones at this step gives the confidence to prepare the histone and DNA mix at the exact proportion].

1. Prepare a solution of 601 DNA plasmid (pGEM3Z-601 from Addgene), H2A-H2B dimers, and dialyzed H3/H4 or CENP-A/H4 tetramer in a buffer (2 M NaCl, 10 mM Tris-Cl pH 8.0, 1 mM EDTA) to make the final volume 200 µl. DNA and histone protein should be gently mixed in the ratio of 10:9 for mononucleosomes. For example, in case of a 40 µg reconstitution, we take 9 µg of H3/H4 tetramers or CENP-A/H4 tetramers and 9 µg of H2A-H2B dimer along with 20 µg of DNA. Then, add 3 µl of protease inhibitor cocktail and incubate on ice for 30 minutes.
2. Gently place the pre-nucleosome mix into a wetted Slide-A-Lyzer cassette (Thermo Fischer, Slide A lyzer 20 K MWCO), and in four sequential steps, gently lifting by the edge with a clean forceps each time, incubate at 4ºC against 500 mL pre-chilled and filtered buffers using a stir-bar at low setting as follows: (a) 2 hours – 1 M NaCl, 10 mM Tris-CL pH 8.0, 1 mM EDTA, (b) 2 hours – 0.8 M NaCl, 10 mM Tris-CL pH 8.0, 1 mM EDTA, (c) Overnight – 0.6 M NaCl, 10 mM Tris-CL pH 8.0, 1 mM EDTA, (d) 2 hours – 0.15 M NaCl, 10 mM Tris-CL pH 8.0, 1 mM EDTA. Then, remove the solution from the dialysis cassette carefully and use immediately.

[Note 2: The 0.6 M NaCl-TE step is critical and should not be diminished since it is the key step at which H2A/H2B dimers will assemble on either side of the pre-nucleosomal tetrasome. The total volume should remain relatively unchanged upon extraction from the dialysis cassette. Loss of material is obvious by severe depletion of the reconstitute volume. If cloudiness is apparent in the tube even after the chromatin is warmed up to room temperature, this is usually indicative of a high histone to DNA ratio, resulting in chromatin aggregates, which can be spun out at high speed].

1. Nucleosome quality evaluation: To determine the quality of the reconstituted nucleosomes, 50% of the reconstitute is digested with Micrococcal nuclease, followed by proteinase K digestion and phenol-chloroform extraction of DNA fragments. The DNA fragments are subsequently analyzed by high-resolution capillary electrophoresis (BioAnalyzer). A high-quality nucleosome ladder should have non-smeary multiples of 150 bp (or ~120 bp for most CENP-A variants), which informs on the quality and uniformity of the reconstitution chromatin arrays. Examples of high-quality histones and subsequent nucleosome reconstitution are provided in Figure 1(b). Chromatin should not be frozen but can be stored at 4ºC for up to 48 hours. In our hands, we have observed that freezing chromatin results in irreversible aggregates as visualized by AFM (Figure 1(c)). Furthermore, spinning the sample in a tube or washing the sample on mica does not reduce the aggregation. We recommend performing MNase analysis in parallel to performing AFM analysis within a few hours after the reconstitution is completed, but certainly not past 24 hrs post-reconstitution. If one chooses, an alternative method is to examine the native nucleosome fragments after MNase treatment without deproteinizing the samples, on native gels. These gels are traditionally run as 0.5% gels in 0.5X TBE at 4°C at low current for several hours. On a native gel, the mono-nucleosome will correspond to ~250–300 bp equivalent to the DNA ladder [42].

### Native chromatin-immunoprecipitation and Western blotting

HeLa cells were grown in DMEM (Invitrogen/ThermoFisher Cat #11965) supplemented with 10% FBS and 1X penicillin and streptomycin cocktail. N-ChIP experiments were performed without fixation. After cells were grown to ~80% confluency, they were harvested as described [43]. For best results for chromatin preparation for AFM the pellet that is obtained after each spin-down during the nuclei extraction protocol [42] is broken up with a single gentle tap. Nuclei were digested for 6 minutes with 0.25 U MNase/mL (Sigma-Aldrich cat #N3755-500UN) and supplemented with 1.5 mM CaCl_2_. Following quenching (10 mM EGTA), nuclei pellets were spun down, and chromatin was extracted gently, overnight in an end-over-end rotator, in a low salt solution (0.5X PBS; 0.1 mM EGTA; protease inhibitor cocktail (Roche cat #05056489001)). N-ChIP chromatin bound to Protein G Sepharose beads (GE Healthcare cat #17-0618-02) was gently washed twice with ice-cold 0.5X PBS and spun down for 1 minute at 4ºC at 800 rpm. Following the first N-ChIP, the unbound fraction was used for the sequential N-ChIP. N-ChIP experiments were performed with Western analyses that were done using LiCor’s Odyssey CLx scanner and Image Studio v2.0.

### AFM sample preparation and scanning in-buffer conditions

(1) Freshly prepare 1-(3-aminopropyl) silatrane (APS)-treated mica (deposit 50 µl 166 µM APS aqueous solution on the mica disk substrates for 30 min, followed by 5 mL wash with ultra-pure water and dry under nitrogen stream) just before each experiment. Dilute reconstituted chromatin sample in 0.5X PBS, 2 mM MgCl_2_ buffer to a final concentration of ~0.01 μg/ml. Deposit 8–10 μl of the diluted chromatin sample at the center of the APS-mica surface. The ends of the pipette tips should be cut to minimize shear flow damage to the chromatin sample. Cover the sample with a sterile petri dish lid to protect it from dust, and incubate for 10 min at RT. Then, rinse the sample with 400–600 μl of the same buffer, dripping 3–4 drops at a time and gently shaking it while holding the sample with tweezers. For imaging in air, rinse the sample with 400–600 μl Milli-Q water, and dry with a gentle flow of argon/nitrogen gas.

To measure the effective elastic moduli of nucleosome samples, we used both the CypherS Asylum (Oxford Instruments) and the MultiMode-8 Nanoscope (Bruker) employing different AFM modes in order to get the most robust comparison between control and experimental samples. In all cases, reconstituted chromatin was first imaged in air by tapping mode AFM to check the sample quality (Figure 1(d–f)) and to establish an appropriate concentration of nucleosomes on the mica substrates (Figure 1(d–f)). For imaging in air, we generally used OTESPA or FESP levers (Bruker).

**Figure 2.**
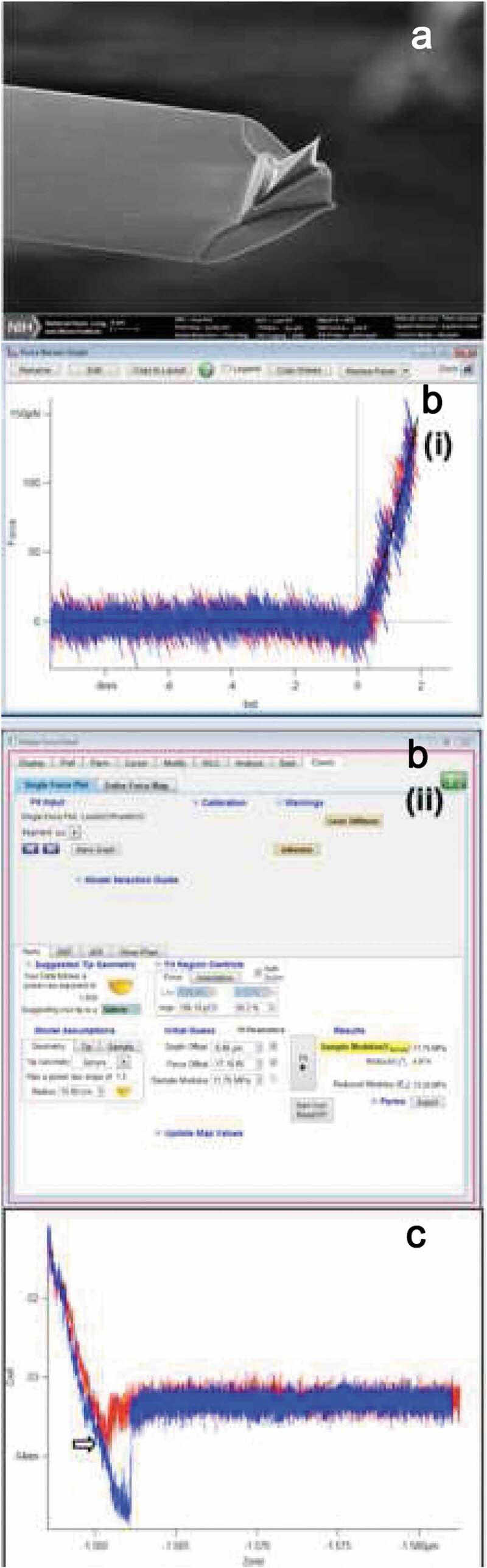
(a) SEM image of Biolever mini cantilever from Olympus, (b) (i) and (ii) showing a screenshot representing indentation vs. force profile for a single nucleosome particle on APS treated mica under 0.5X PBS buffer, 2 mM MgCl_2_, the approach force curve (red) is fitted with the hertz model using spherical geometry, (c) A force vs. distance profile where the approach force curve (red) has an adhesion peak.

(2) For the AFM CypherS Asylum microscope, prepare the samples at the desired concentrations, mount on the instrument stage, and scan under buffer in tapping mode (contact mode imaging is not suitable for such biological samples, because the shear forces will damage the sample) using cantilevers with a low spring constant, e.g. Biolever mini from Olympus and MSNL from Bruker with spring constant in the range of 0.01–0.1 N/m. These probe tips are ~2–10 nm in a radius of curvature which is close to the dimensions of nucleosomes (Figure 2(a)). The frequency for biolever mini levers is in the range 20–26 kHz, which is a high enough frequency for achieving high-resolution images in buffer, in comparison to other available probes for in-fluid imaging. We typically use a 100 µl buffer droplet of the chromatin deposited mica surface to immerse the cantilever. In buffer, while approaching, setpoint for scanning should be kept at approximately half the free oscillation amplitude. Scanning should be performed gently, at low speed (0.5–1 line/s) to prevent sample damage, and images captured with dimensions 2 µm × 2 µm initially with resolution 512 × 512 or higher, then zoomed in to 1 µm × 1 µm or 500 nm × 500 nm to magnify an area of interest.

[Note 3: i) the mica should be strongly attached on to the metal stub; ii) use mica that is not extremely thin; iii) use room temperature buffer to minimize drift during imaging; iv) check the frequency of the cantilever under buffer frequently to make sure you are at the right frequency peak].

### AFM nanoindentation force spectroscopy under buffer conditions (for Cypher)

(1) In order to calibrate the AFM probe for force spectroscopy, before immersing the AFM probe in the buffer, use GetReal automated probe calibration (free application for all Cypher Asylum AFMs). Here, the tip never touches the sample, minimizing the risk of damage or contamination. The inverse optical lever sensitivity (InvOLS) and spring constant (k) are both calibrated in one step.

For other AFMs, the probe calibration methods are straightforward and can be found in the instrument manual in detail. Briefly, the deflection sensitivity has to be measured from the contact region of a force curve taken on a hard substrate, e.g. freshly cleaved mica, followed by thermal tuning of the cantilever to estimate the spring constant of the lever. It is always recommended to use new tips for calibration and it is good practice to repeat the calibration a few times to check the deflection sensitivity value on the hard substrate at different locations.

(2) Engage the tip in AC mode now and start scanning the sample in buffer, once you get a decent quality image of the chromatin sample, zoom in to 1 µm × 1 µm. Measure the dimensions of the nucleosomes and ensure the quality of the reconstituted material. Usually, the height values of in vitro reconstituted H3 and CENP-A nucleosomes are 4–5 nm under buffer conditions. The diameter should be spherical at ~12–14 nm. Now, you are ready to switch to ‘contact’ mode for acquiring force curves.

(3) First, set the trigger force as 150–200 pN. For the Cypher AFM system, we have found that ~200 pN force gives stable measurements. Then, acquire force curves on a nucleosome particle by using ‘pick a point’ tool (similar tools are available in all commercially available AFMs). Repeat this for a number of times at different points on a nucleosome as well as other nucleosomes in the same scan area. Similarly, Force maps can also be acquired on a particular area containing nucleosomes. Next, fit the acquired force curves with ‘Hertz’ model with a specific tip geometry (a screenshot of Cypher AFM software showing fitting a force curve with Hertz model (spherical geometry) is presented in Figure 2(b)). Nucleosomes were indented not more than 1.5 nm. If the approach force curve contains adhesion peaks (arising from stickiness of the sample), that force curve should not be considered for subsequent analysis since Hertz model can only be applied when the adhesion force is much smaller than the maximum force applied (Figure 2(c)). In order to plot the data points together from multiple experiments and to generate the histograms, the ascii files can be opened and plotted using the ‘origin’ software(https://www.originlab.com/).

### PeakForce tapping – quantitative nanomechanical mapping (PFT-QNM)

For the MultiMode-8 Nanoscope by Bruker, the most appropriate AFM modality is the PeakForce Tapping – Quantitative Nanomechanical Mapping (PFT-QNM). In this AFM mode, the cantilever is excited at frequencies well below their resonance (typically, 0.5 to 2 kHz, but lower and higher frequency capabilities are also available) and a force curve is acquired at every oscillation cycle. The instrument software then constructs a topographical image by estimating the contact point at every oscillation cycle. Simultaneously, the individual force curves are fitted with appropriate physical models to obtain an estimate of the effective elastic modulus of the sample. It is recommended to use sharp silicon nitride probes with stiffness of around 0.1 N/m (e.g. MSNL-E, Bruker whose radius of curvature is on the order of 3 nm). The procedure will result in very high-resolution topography and elasticity mapping. For the sharp probes recommended above, we use the Sneddon model for conical probe. The maximum force (Peak-Force) at each oscillation cycle should be maintained below 100 pN and the oscillation amplitude should be reduced to 10–15 nm at oscillation frequency of 1 kHz. In all cases, the maximum indentation should be limited to 1–1.5 nm beyond which, damage to the nucleosomes was observed.

Initially, a larger area is scanned in this way to obtain an overview of the sample before zooming in to 250–500 nm^2^ area for high-resolution QNM imaging (1–2 nm/pixel). The acquired data contain a force curve for each pixel in the topography image. One may use the automatic analysis provided by the instrument software to analyze the force curves and plot an elasticity map of the sample or one may export the force curves for off-line, custom curve-fitting. One issue with the QNM force curves is that they can be noisy and one may need to resort to careful, custom curve-fitting to ensure correct localization of the contact point and the extracted elasticity modulus. We found this to be the best method for reliable parameter estimation and we only need to analyze a small subset of the total force map by choosing force curves along short line segments running across single nucleosomes. Typically, there will be less 10 curves across each nucleosome and those curves have to be selected carefully using the topography as a guide.

**Figure 3.**
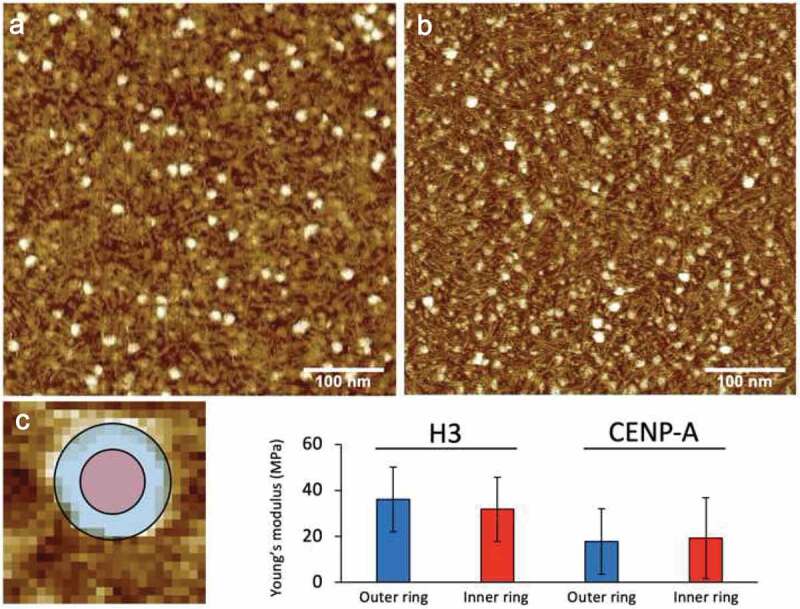
Measurement of Young’s modulus on mono-nucleosomes; representative images of (a) H3 and (b) CENP-A mono-nucleosomes on mica. (c) Young’s modulus was measured across individual H3 or CENP-A mono-nucleosomes to assess whether a nucleosome particle is uniformly elastic, indeed both the inner (red) and outer (blue) ring showed similar Young’s modulus values.

As an example, mononucleosomes were reconstituted on 187 bp of 601 Widom sequence using either canonical H3 or the CENP-A variant. The mica surfaces were modified with 0.5 mM APS and we used MSNL-E cantilevers (Bruker) that were individually calibrated with the thermal noise method provided with the instrument software. Force curves across individual nucleosomes were exported and analyzed with custom written (MATLAB) fitting software that models the probe as a cone (Sneddon). The results from H3 and CENP-A mononucleosomes are shown in Figure 3.

### *AFM nanoindentation spectroscopy of* ex vivo *purified chromatin*

**Figure 4.**
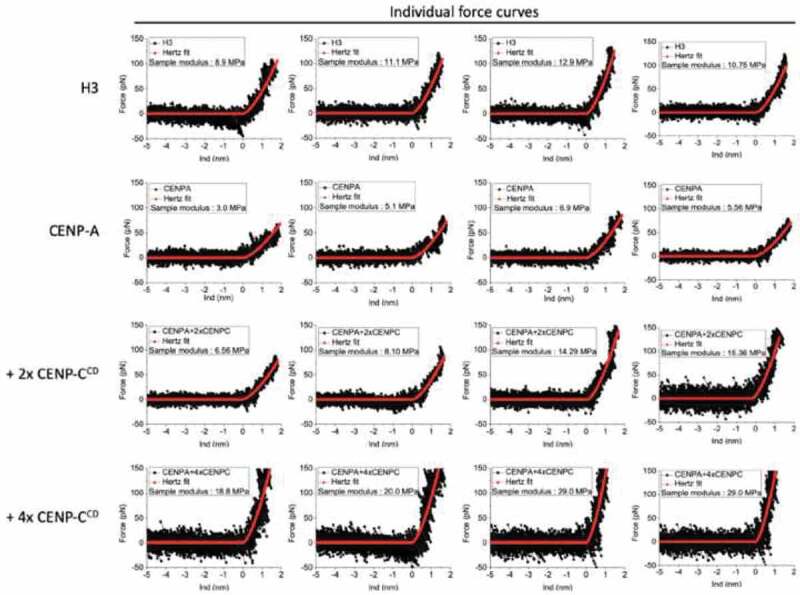
Examples of force curve measurements on nucleosomes in an array: four representative force curves for H3 nucleosomes, CENP-A nucleosomes, CENP-A nucleosomes with twofold excess CENP-C^CD^ fragments, and CENP-A nucleosomes with fourfold excess CENP-C^CD^ fragments are shown. (Data reproduced with permission from Melters *et al* 2019 PNAS [18]).

Recently, we successfully used the above-described protocol to measure the elasticity of CENP-A, H3 and CENP-C associated CENP-A chromatin (Figure 4 and Table 1) [18]. Next, we analyzed bulk chromatin purified from HeLa cells.

**Table 1.** Nanomechanical force spectroscopy investigations on H3 and CENP-A nucleosomes and CENP-A nucleosomes bound by CENP-C^CD^ (*in vitro*), bulk and CENP-C N-ChIP samples (ex vivo).

Name of sample	N	Height (nm)	Diameter (nm)	Young’s modulus (MPa)
H3 mononucleosome	5	5.2 ± 0.5	11.3 ± 1.2	35.4 ± 13.9
CENP-A mononucleosome	4	5.7 ± 0.5	11.7 ± 2.3	18.5 ± 15.6
H3 nucleosome array	48	3.8 ± 0.3	14.0 ± 1.2	11.3 ± 4.1
CENP-A nucleosome array	46	3.7 ± 0.3	13.7 ± 1.0	5.8 ± 3.0
CENP-A + 2X CENP-C^CD^	48	4.1 ± 0.5	13.5 ± 0.9	9.4 ± 5.8
CENP-A + 4X CENP-C^CD^	50	4.1 ± 0.6	14.0 ± 1.2	15.2 ± 10.5
Bulk chromatin	13	5.4 ± 0.5	14.3 ± 0.8	16.1 ± 5.5
CENP-C complex (CENP-C N-ChIP)	5	8.3 ± 1.8	41.9 ± 6.8	36.5 ± 10.5

N, number of particles measured in fluid (0.25X PBS + 2 mM MgCl2) (*in vitro* experimental data are reproduced with permission from Melters et al. 2019 PNAS [18]).

**Figure 5.**
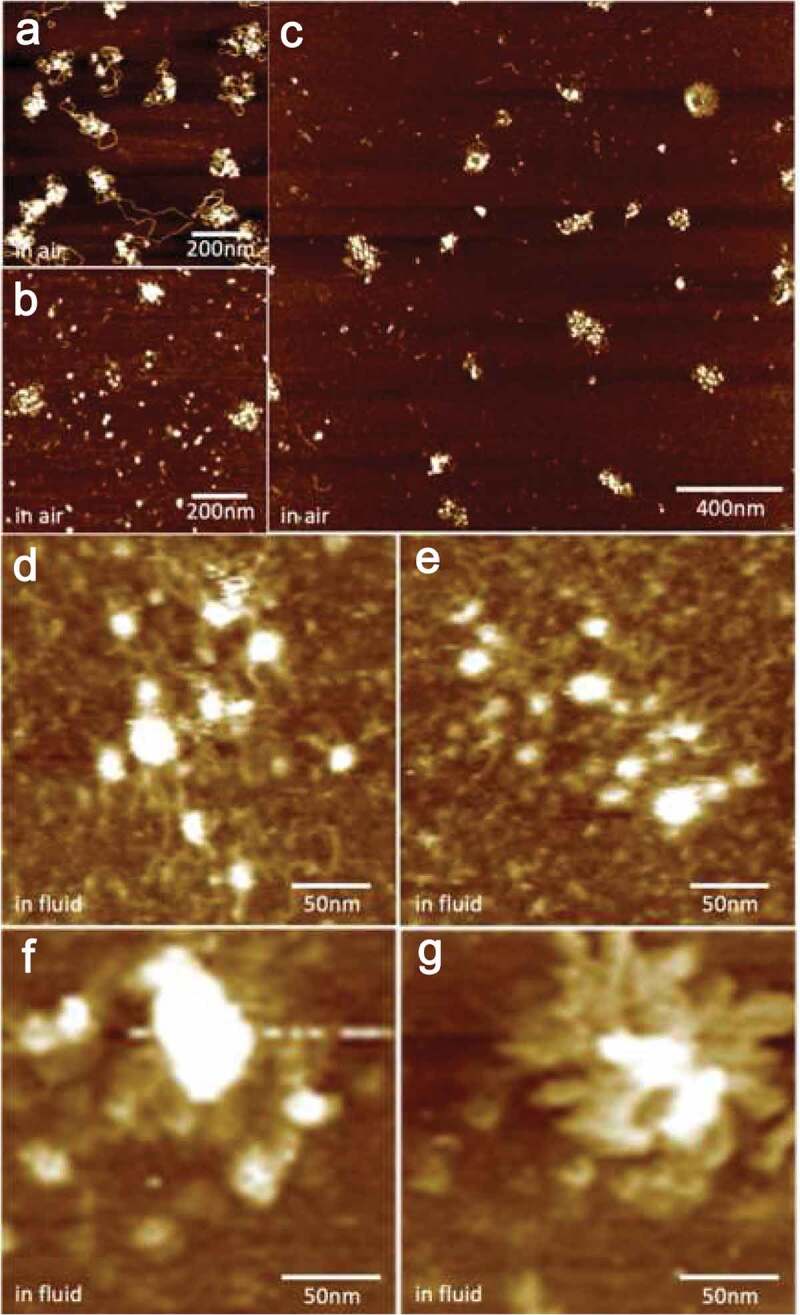
In air AFM images of CENP-A nucleosome arrays from (a) *in vitro* reconstitution, (b) and *ex vivo* CENP-A ChIP, and (c) Bulk chromatin extracted from HeLa cells. In fluid (0.25X PBS + 2 mM MgCl_2_) AFM images from (d and e) Bulk chromatin and (f and g) CENP-C N-ChIP.

We note these experiments are non-trivial principally due to the complex nature of the sample. There are a number of factors to be particularly noted for successful operation with an *ex vivo* sample. First, in *in vitro* experiments, the precise composition of your sample is regulated, including the concentration (Figure 5(a)), whereas *ex vivo* samples inherently contain impurities derived from the cell (Figure 5(b)). Second, these samples tend to not adhere strongly to the APS-modified mica surface, which is an important prerequisite to get a good quality AFM image in buffer conditions. Despite all these caveats, native samples represent true biological substrates, and therefore are worthy of investigation in order to validate and functionally dissect properties observed *in vitro*. To overcome these problems, we tested the stability of bulk chromatin in different buffer conditions by varying the salt concentrations. We also tested how well bulk chromatin adheres to the mica surface by using various APS concentrations. From these experiments, we have observed that a twofold increase in APS concentration (deposit 50 µl 332 µM APS aqueous solution) and a buffer solution containing sub-physiological concentration of NaCl (30 mM) stabilize *ex vivo* chromatin samples on the mica.

While probing mechanical characteristics of a chromatin fiber, maintaining physiological salt concentration and the presence of Mg^2+^ ions are critical in order to restrict rotation of the nucleosomes within the fiber. The first report on mechanical properties of higher-order chromatin structure by the Bustamante lab [44], who showed a transition from a folded chromatin fiber to an unfolded fiber when lowering the salt concentration to 40 mM (without MgCl_2_). Furthermore, they show that these protein-fiber interactions are salt-dependent and disappear in low salt concentration (~5 mM) [44]. Many reports with optical and magnetic tweezers showed experiments performed at low salt and in absence of Mg^2+^, i.e. conditions where higher-order folding can be expected to be severely affected yielding variable results [45–51]. Sub-physiological salt solutions are also often used for fluid AFM operation to get stable imaging of nucleosomes [52,53]. The nucleosomal stability also depends on the core particle concentration [50,51]. Indeed, a native nucleosomal state consisting of two copies of H2A, H2B, H3, and H4 are stably bound to DNA over a broad range of salt concentration (2–750 mM) in a nucleosome density-dependent manner [54,55].

For our *ex vivo* experiments, MgCl_2_ concentration is always maintained at 4 mM along with sub-physiological concentration of NaCl (30 mM) to stabilize chromatin. We first visualized the bulk chromatin sample by tapping mode AFM in air to check the quality of the sample (Figure 5(c)). If we could see at least a few nucleosome arrays within 2 µm × 2 µm scan area and the heights of the nucleosomes are close to 2.5 nm, then we moved to imaging the sample in buffer conditions. The height and diameter values of bulk nucleosomes (5.4 ± 0.5 nm and 14.3 ± 0.8 nm) in buffer are higher than *in vitro* reconstituted H3 nucleosomes (3.8 nm±0.3 nm and 14.0 ± 1.2 nm) [18] (Figure 5(d, e), Table 1). After imaging in tapping mode in fluid, force curves of bulk nucleosomes were obtained and the Young’s modulus of individual particles was determined. These results demonstrated that the bulk H3 nucleosomes extracted from human cells are slightly more rigid relative to *in vitro* reconstituted H3 nucleosomes (16.1 ± 5.5 MPa vs. ~11.3 ± 4.1 MPa, respectively, see Table 1) [18].

One plausible and exciting explanation for this intriguing increased rigidity *of ex vivo bulk* nucleosomes is the binding of linker histone H1 to the H3 nucleosome. The linker histone is present at half-molar equivalence with canonical nucleosomes in most eukaryotes [56] and is thought to be bound to every other nucleosome [57]. Alternative sources might be the intrinsic heterogeneity of canonical nucleosomes, arising from varying DNA sequences, DNA modifications, histone modifications, nucleosome binding proteins, and active processes upon the chromatin fiber. Still, the range of elasticity for bulk nucleosomes falls well within the range observed *in vitro*. Thus, these experiments provide proof-of-principle that our adaptation of nanoindentation analysis can be applied to chromatin extracted from human cell nuclei.

**Figure 6.**
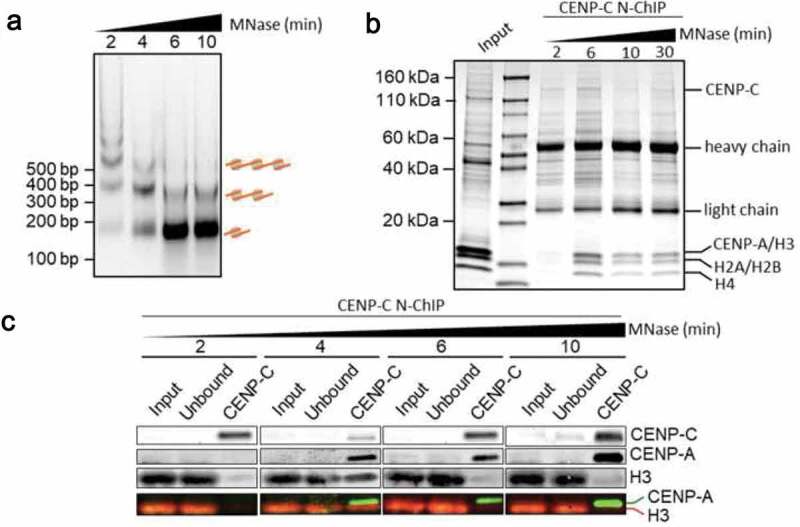
(a) DNA products followed a time course of MNase digestion were resolved on a 1% agarose gel. (b) CENP-C N-ChIP samples obtained from a MNase digestion time course were resolved on 4–20% SDS Page and stained with total protein. (c) Western blot analysis of CENP-C ChIP following a MNase time course showed that after 6-minute MNase digestion, CENP-C ChIP almost exclusively pulls down CENP-A nucleosomes.

To further study the mechanical characteristics of *ex vivo* obtained nucleosomes bound to specific partners, we extracted chromatin from HeLa cells as noted above [18,42]. Chromatin was digested with MNase and extracted for 6–12 hours at 4°C [42]. To quantify the extent and quality of chromatin thus released, a 1.5% agarose gel should be run with isolated DNA fragments. A good quality digestion and chromatin purification should not be smeary but should possess a striking ladder of fragments with multiple of a unit repeat as first observed by Hewish and Burgoyne [58]. A smeary ladder usually reflects degraded chromatin, as does large precipitation in the solvate. The extent of the ladder reflects the length of chromatin fragments released from the MNase digest. Next, to purify a specific chromatin fraction, immunoprecipitation (ChIP) is performed on these samples. In our case, to isolate the kinetochore bound centromeric chromatin, we used a validated antibody against the inner kinetochore protein CENP-C. The efficiency of the ChIP was further validated by western blotting after SDS-PAGE of the proteins in the IP (Figure 6). The experiment that follows requires validation that the proteins of interest are indeed enriched in the IP and that the chromatin is of high quality. Care should be used to maintain the chromatin in physiological buffers at all times, with a minimum of 2 mM MgCl_2_ and 75 mM NaCl containing buffer, plus protease and nuclease inhibitors to prevent degradation. In our hands, chromatin stored for more than a few days at 4°C is usually of poorer quality than freshly prepared chromatin. Indeed, we strongly recommend working through the protocol in one flow, from nuclear extraction all the way to AFM analysis within a period of 48 hours.

We next adapted our nanoindentation protocol to analyze the mechanical characteristics of purified CENP-C complexes (CENP-C ChIP). First, upon visual analysis, we observed tall structures (8.3 ± 1.8 nm) that associated with nucleosomes (Figure 5(f, g)). Here, we find that Young’s modulus values of the CENP-C complexes are ~36 MPa, more than twice as rigid as bulk H3 canonical nucleosomes (16 MPa) (Table 1). We previously reported that recombinant CENP-A nucleosomes *in vitro* are rather elastic at 6 MPa, relative to H3 nucleosomes (~11 MPa), or to recombinant peptide-CENP-C^CD^ bound to CENP-A nucleosomes (~15 MPa) (Table 1). These data suggest that the CENP-C complex is rigid (~36 MPa) relative to the native chromatin polymer.

More experiments using antibodies against and mutations of other kinetochore proteins will be needed to decipher which of the 40+ members of the kinetochore impart this rigidity. More intriguingly, these data lead us to speculate that rigidity is an emergent and reversible property of an inter-connected Boustrophedon-like network [59] proposed by Bill Earnshaw and colleagues, and the spring-like stretchable nature of the inner kinetochore chromatin fiber proposed by Kerry Bloom and colleagues [10].

## Future applications

Macromolecular structures produce mechanical force properties such as elasticity, viscosity, and thermal motion, which are important [59]. Chromatin fibers possess unique mechanical properties that contribute to its function [60]. Herein, we describe adaptations of our recently developed single-molecule nanoindentation tools [18] applied to bulk chromatin purified from human cells. Interestingly, we found that bulk nucleosomes were substantially more rigid compared to *in vitro* reconstituted H3 nucleosomes. This observation can be caused by several factors. In the nucleus, there is a ratio of 0.5 linker histone H1 for every core particle [57]. H1 binds at the nucleosome dyad, fixing the entry and exit DNA strand [61,62]. It is conceivable that this interaction rigidifies nucleosomes, although this has never been formally demonstrated. In contrast to *in vitro* reconstituted nucleosomes, extracted nucleosomes are diverse in their histone composition and post-translation modification repertoire. Indeed, it has long been recognized that nucleosome arrays can fold into 30-nm fibers *in vitro* [63–65], but several recent studies using cryoEM tomography on human nuclei observed chromatin chains varying in size between 5 and 24 nm [66–68]. It is also possible that other chromatin-binding proteins and RNAs that associate with chromatin alter its mechanical nature in a manner hitherto not know. Altogether how these factors change the mechanical response of the chromatin fiber, and how this physically impacts chromatin dynamics is an exciting avenue which has not been explored in the field.

Second, we applied our method to CENP-C complexes purified from human cells. In this case, we observe that these large complexes are significantly more rigid compared to nucleosomes from bulk chromatin. In our *in vitro* study (Melters et al. 2019) a CENP-C fragment rigidified CENP-A nucleosomes, which in human cells correlated with reduced RNA polymerase 2 occupancy at centromeric chromatin when CENP-C was overexpressed. Together these data provide a clue that the kinetochore might form on rigidified CENP-A chromatin, thereby altering chromatin accessibility. It will be interesting to unravel how nucleosome binding proteins modify the individual nucleosomes and how these modifications change the accessibility of the chromatin fiber. Indeed, changes in chromatin accessibility have been observed in both cancers and aging [69–72]. We, and others, have documented that in many types of human cancers, CENP-A is overexpressed and ectopically localized to neocentromeric breakpoints. One such locus, we showed previously, includes the 8q24/Myc region long-associated with genomic instability [73]. Since, CENP-A nucleosomes are highly elastic compare to H3, it might be possible, that, in the cancer genome they get accumulated to inappropriate sub-telomeric locations as an unexpected mechanical outcome; alternatively, the formation of weak kinetochores as such location might make them susceptible to DNA damage because of the unexpected rigidity imparted by the kinetochore complex. These unexplored changes in histone content and quality in diseased tissue should provide a rich area of investigation.

Other applications of this methodology apply to the evolution of chromatin mechanics. Although all centromeres facilitate faithful chromosome segregation, the underlying DNA and centromeric and kinetochore proteins are fast evolving [74,75]. Despite these evolutionary changes, the unique chromatin structure of centromeres as seen by light microscopy appears generally well conserved. Therefore, it is of interest to study how conserved mechanical properties of centromeric chromatin are across species. This will help to understand how these epigenetic strategies evolve, and their contribution to biological functions.

How genomic DNA is made accessible at the right time, in the right cell, and in the right order is vital for organismal survival. The molecular composition of chromatin, most notably histone variants, PTMs, and chromatin binding factors contribute to the mechanobiological properties of the chromatin fiber. In addition, DNA topology induced by either molecular machineries, sequence composition, or invading RNAs might also impact the biomechanical properties of nucleosomes and the chromatin fiber. Therefore, as detailed in this method, analyzing biomechanical features of nucleoprotein complexes at single-molecule resolution provides a broadly applicable experimental tool with which one can decipher a fascinating new layer of eukaryotic genome regulation [76].
